# Evaluation of sexual dysfunction in patients with schizophrenia:A descriptive cross-sectional study

**DOI:** 10.1192/j.eurpsy.2023.2259

**Published:** 2023-07-19

**Authors:** K. Mahfoudh, A. Ouertani, W. Boumaiza, A. Aissa, U. Ouali, R. Jomli

**Affiliations:** 1 “A” Department, Razi Hospital, Manouba, Tunisia

## Abstract

**Introduction:**

Schizophrenia is a common chronic mental illness (1% of the general population) classified by the World Health Organization in the group of the 10 most disability causing diseases.

Despite its impact on social and relational functioning and the alteration of the quality of life, the sexuality of these patients is not always explored.

**Objectives:**

Evaluate the sexuality of patients with schizophrenia by comparing men and women followed in the "A" psychiatry department of Razi Hospital in Tunisia.

**Methods:**

A descriptive cross-sectional study was conducted with 50 stabilized patients (25 men and 25 women) suffering from schizophrenia.

The research for sexual dysfunctions was carried out with the ASEX scale (Arizone Sexual Experience Scale) and CSFQ-14 (Changes in sexual functioning questionnaire) in their French version.

A correlation was used between these two scales in order to guarantee results’ conformity.

**Results:**

The sexual activity rate was 64.0% at the time of the study and 88.0% over a life span. These sexual activity rates were comparable between the two sexes.

The overall rate of sexual dysfunction was 68.7% of sexually active patients at the time of the study and concerned 72.2% of men and 64.3% of women, with no difference according to gender.

The different sexual dysfunctions were equally present in men and women, except for the dimension disorder: desire/interest, desire/frequency and arousal, which were more frequent in women.

**Conclusions:**

The sexual aspect in patients followed for schizophrenia remains neglected by clinicians. It deserves better attention in order to optimize the overall care of patients and improve their quality of life.

**Disclosure of Interest:**

None Declared

